# Point prevalence, lab-based survey of antimicrobial resistance in referral and regional hospitals in Oman

**DOI:** 10.1186/2047-2994-4-S1-P128

**Published:** 2015-06-16

**Authors:** B Zayed, K AL Harthy, B AL Abri

**Affiliations:** 1 Infection Prevention & Control Department, Ministry of Health, Oman, Muscat, Oman

## Introduction

Anti-Microbial Resistance (AMR) is both a clinical challenge and a public health threat as AMR infection worsens patient clinical outcome and burden of health systems. Cost of treatment, lengths of hospital stays, and morbidity and mortality increased. Few studies have described the prevalence of antimicrobial resistance in Oman, but those studies are limited to either one hospital or one organism.

## Objectives

This study aims to assess the prevalence of antimicrobial resistance among the tertiary and secondary care governmental hospitals in Oman.

## Methods

This is a point-prevalence survey using the World Health Organization (WHO) tool [1] conducted 16-22 March 2014. 11/15 (73.3%) of the referral and regional hospitals of the country participated. Microsoft Excel used to collect data on five AMR organisms; *Methicillin-Resistant Staphylococcus aureus* (*MRSA*), *Vancomycin-Resistant Enterococci* (*VRE*), *Extended-spectrum β-lactamase* (*ESBL*) *producing Enterobacteriaceae*, *Carbapenem-Resistant Enterobacteriaceae* (*CRE*), *and Multi-Resistant Acinetobacter spp* (*MRAB*)*.* SSPS software used to describe the overall results.

## Results

254 bacterial isolates have been identified. 90 (35.4%) are Gram positive {*Staphylococcus aureus* (13 isolates, 14.4%), Enterococci spp (11, 12.2%) and others (66, 73.3%)}, while 164 (64.6%) are Gram negative {*Enterobacteriaceaespp*. (120 isolates, 73.2%), *Acinetobacter spp*. (10, 6.1%), and others (34, 20.7%)}. 47/254 (18.5%) isolates have AMR feature (either *MRSA*, *VRE*, *ESBL*, *CRE or MRAB*)*.* 6 (60%) are *MRAB* out of the 10 total *Acinetobacter* isolates. Among the 73 isolates of *E. coli*, 24 (32.9%) isolates are *ESBL* while no *CRE* identified. 10 (27%) *ESBL* isolates recognized among the 37 *Klebsiella pneumonia*e and 2 (5.4%) are *CRE*. 4 *MRSA* isolates (30.8%) identified among the *Staphylococcus aureus.* One sample (9%) is *VRE* among the *Enterococci spp*.

## Conclusion

The prevalence of resistance is higher in *Acinetobacter*, *E.coli*, *MRSA*, *Klebsiella*, and *Enterococci* respectively. This study is the first prevalence survey that covers multiple organisms and many hospitals in Oman and provides important baseline. Capacities are available in the participating hospitals to identify AMR organisms. Continual and national surveillance is the optimal goal for the accurate and generalized data on AMR.

## Disclosure of interest

None declared.

